# Qualitative Document Analysis of Patient’s Healthcare Trajectory With and Without Palliative Care

**DOI:** 10.1093/geroni/igab046.3220

**Published:** 2021-12-17

**Authors:** Melissa Steffen, Kenda Stewart Steffensmeier, Bruce Alexander, Maresi Berry-Stoelzle

**Affiliations:** 1 VA Office of Rural Health, Veterans Rural Health Resource Center-Iowa City (VRHRC-IC), Iowa City, Iowa, United States; 2 Iowa City VA Health Care System, Iowa City, Iowa, United States; 3 Iowa City VA Medical Center, Iowa City, Iowa, United States

## Abstract

Palliative care is important to the care of seriously ill patients to support the patient and family. Palliative care is often timely in the inpatient setting, but delayed in outpatient care, leading to missed opportunities. Identifying when to engage patients with palliative care in outpatient settings has been challenging. As part of a larger quality improvement project to increase access to palliative care, a qualitative sub-study was completed to identify missed palliative care engagement opportunities in patient’s healthcare trajectories. A document analysis of patients notes from a convenience sample of 20 recently deceased patients who received care within the Veteran Affairs healthcare system (VAHCS) was completed. Patients were sorted into four categories that emerged from initial analysis: cancer/palliative, non-cancer/palliative, cancer/non-palliative, and non-cancer/non-palliative. Two qualitative analysts reviewed the notes, paying particular attention to notes preceding or following seminal healthcare events. Patients in the cancer/non-palliative category were more likely to decline preventive care, engage less with the VAHCS health care or only interacted with the VAHCS for specific needs (e.g., determine VA health benefits). Similarly, non-cancer/non-palliative care patients were more likely to use a mix of VAHCS and outside healthcare, with inpatient care occurring outside of the VAHCS. For non-palliative care patients, seminal healthcare events were less likely to occur in the VAHCS. Thus, identifying opportunities to engage patients with palliative outside of seminal healthcare events may be important to increasing patient access within the VAHCS.

